# Sporadic Insulinoma as a Rare Cause of Recurrent Hypoglycemia in Children

**DOI:** 10.1155/2017/4756793

**Published:** 2017-04-20

**Authors:** Hedyeh Saneifard, Ahmad Khaleghnejad Tabari, Maryam Kazemi Aghdam, Mohadese Musavi Khorshidi, Ali Sheikhy

**Affiliations:** ^1^Pediatric Endocrinology and Metabolism, Shahid Beheshti University of Medical Sciences, Tehran, Iran; ^2^Pediatric Surgery Research Center, Shahid Beheshti University of Medical Sciences, Tehran, Iran; ^3^Pediatric Pathology Research Center, Shahid Beheshti University of Medical Sciences, Tehran, Iran; ^4^Mofid Hospital, Shahid Beheshti University of Medical Sciences, Tehran, Iran; ^5^Tehran University of Medical Sciences, Tehran, Iran

## Abstract

Insulinoma is a rare pancreatic tumor in children and adolescents. As a result of insulin hypersecretion, signs and symptoms are more commonly consequences of the pathophysiologic responses to hypoglycemia. According to rarity of this tumor in children and nonspecificity of clinical presentations, diagnosis of insulinoma in this group of patients is usually delayed. Early diagnosis is very important for preventing neurologic damage. In this case report, we present the case of a 10-year-old boy with signs and symptoms of hypoglycemia and final diagnosis of insulinoma.

## 1. Introduction

Insulinoma is a very rare tumor in children and adolescents. Diagnosis of insulinoma is often delayed in this group of patients because neuropsychiatric signs and symptoms such as confusion, personality change, ataxia, and seizure that occur following hypersecretion of insulin by this tumor and consequent hypoglycemia and neuroglycopenia are commonly seen in a variety of other pediatrics diseases [1, 2]. The incidence of insulinoma is higher in adults and diagnostic criteria are more clearly defined. In children, pancreatic insulinomas are usually solitary and benign tumors. Prompt and early diagnosis of insulinoma is very important because it can cause recurrent hypoglycemia which can consequently cause irreversible neurologic damage. As mentioned before, most insulinomas in children are benign and so can be treated successfully by surgical resection. In this case report, we present the case of a 10-year-old boy who was referred with neuroglycopenic signs and symptoms such as seizure, loss of consciousness, and recurrent episodes of hypoglycemia. Laboratory and radiologic investigations confirmed insulinoma.

## 2. Case Report

**Figure 1:**
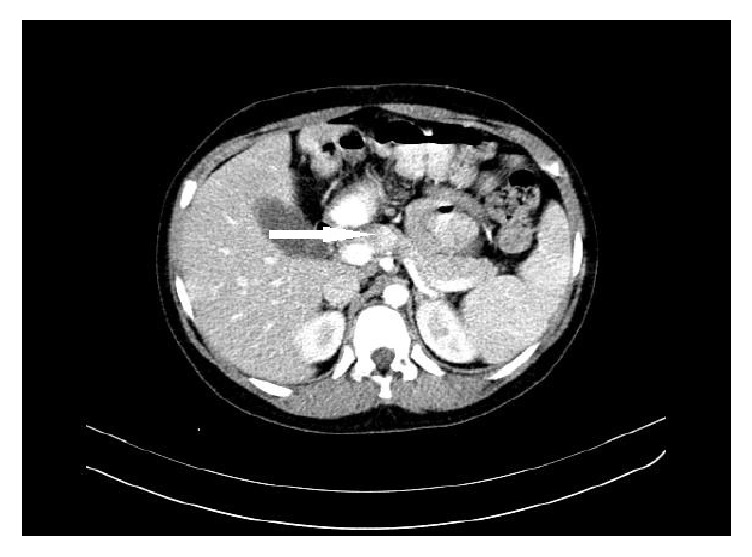
Abdominal CT scan with enhanced mass at the neck of pancreas (arrow).

**Figure 2:**
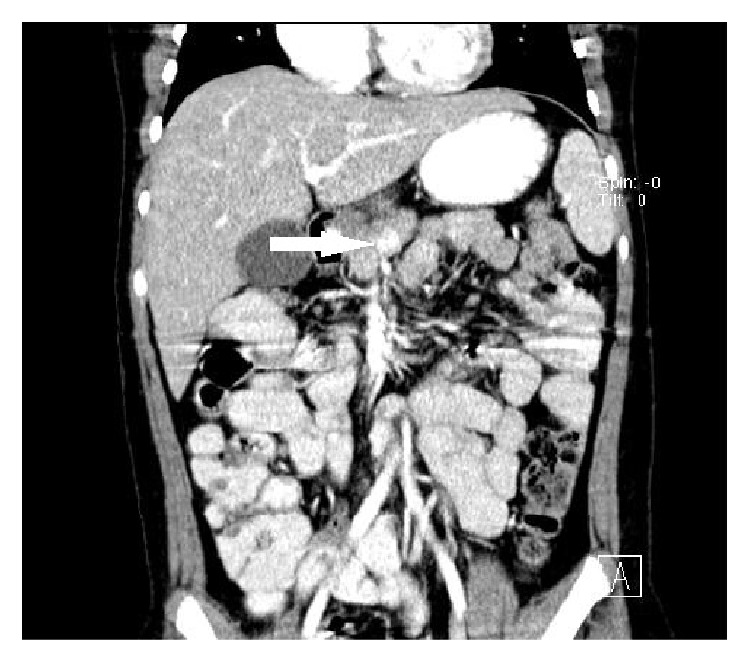
Abdominal CT scan (coronal view) shows intrapancreatic tumor (arrow).

**Figure 3:**
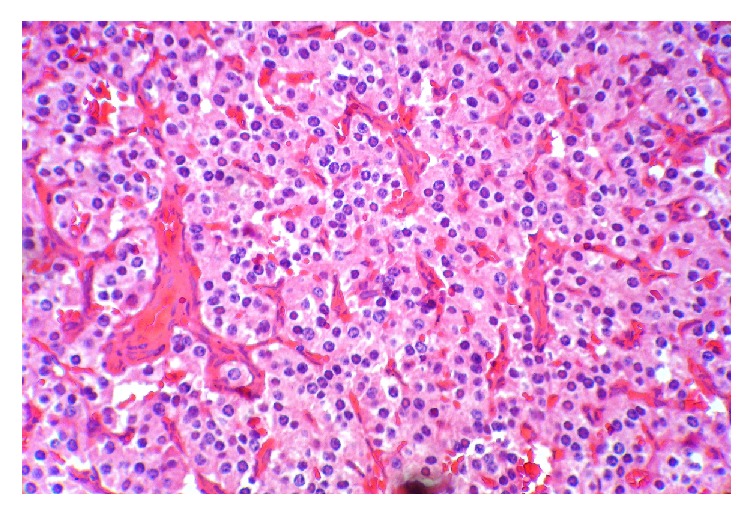
Pancreatic endocrine tumor with nests of uniform cells surrounded by a fibrovascular stroma.

**Figure 4:**
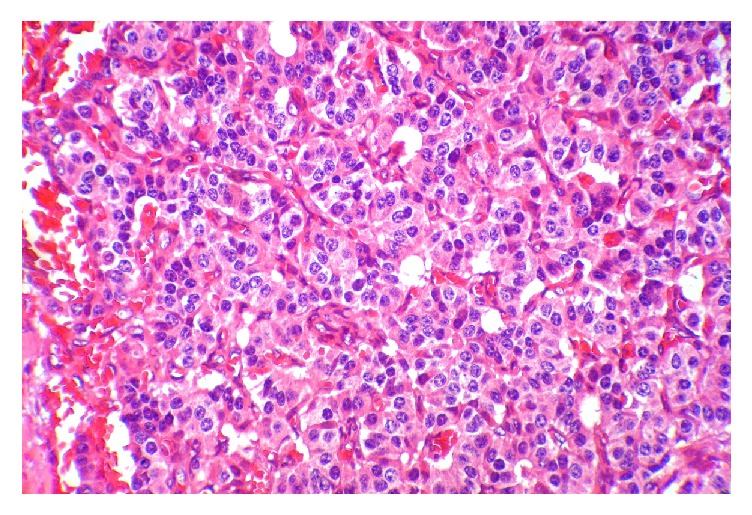
Nuclear features show coarsely clumped, “salt and pepper” chromatin pattern.

**Figure 5:**
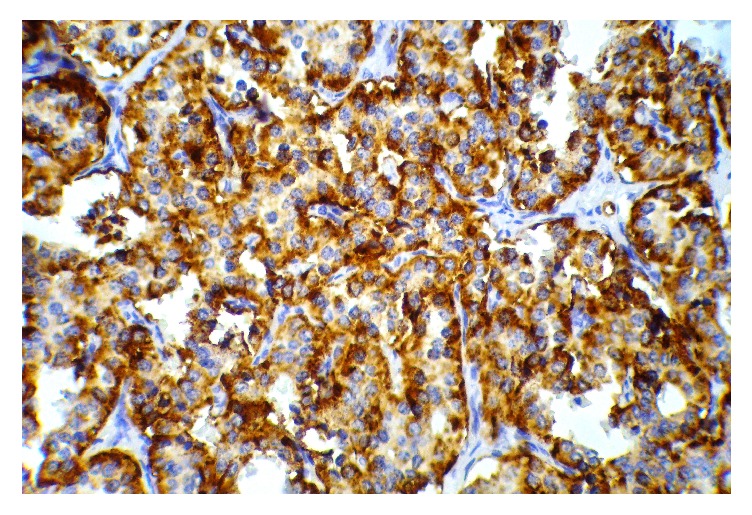
IHC for chromogranin (one of the endocrine differentiation markers) expressed by tumor cells.

A 10-year-old obese boy was referred to pediatrics endocrinology clinic with history of recurrent episodes of generalized weakness and loss of consciousness for six months. He had also multiple attacks of generalized tonic-clonic seizure for one month. He was obese (90th percentile) and tall (75th percentile). There was no positive finding in his past medical and familial history and physical examinations revealed no abnormal findings. The most important finding in initial laboratory investigations was hypoglycemia; blood sugar (BS) was 35 mg/dL. In complementary investigations at the time of hypoglycemia, serum insulin level was 35.8 mIU/L (normal level < 2 mIU/L), C-peptide was 8.9 ng/mL (normal range: 0.15–2.72 ng/mL), urine ketone was negative, serum cortisol was 18.4 *μ*g/dL (normal range: 7–28 *μ*g/dL), serum ACTH was 28.5 pg/mL (normal range: 10–60 pg/mL), and serum GH was 12 ng/mL (normal range: 0–20 ng/dL). In the presence of normal serum GH and cortisol at the time of hypoglycemia, GH and cortisol deficiencies were ruled out and also high level of serum insulin confirmed hyperinsulinism as the cause of hypoglycemia. Serum prolactin level was checked that, according to normal level of this hormone, multiple endocrine neoplasia type I (MENI) was ruled out. With suspicious diagnosis of hyperinsulinism, treatment with diazoxide was started and, according to unusual age of the presentation, spiral abdominal CT scan with intravenous and oral contrast was done. CT scan revealed 7.6 × 13 mm solitary and enhancing lesion at the neck of pancreas in favour of insulinoma (Figures 1 and 2). With clinical diagnosis of pancreatic insulinoma, the patient underwent open subtotal pancreatectomy; pathologic examination of specimen with complementary immune histochemical (IHC) staining confirmed insulinoma as the final diagnosis (Figures 3–5). There was no postoperative surgical and endocrinological complication and serial evaluation of blood sugar revealed normoglycemia. In follow-up of patient in a six-month period, he was completely free of neuroglycopenic signs and symptoms and blood sugar remained in normal range.

## 3. Discussion

Hypoglycemia has different etiology in children but insulinoma is final diagnosis in only minority of them. Insulinoma is the most common neuroendocrine tumor of pancreas. Most insulinomas are solitary and sporadic, although about 10% are associated with MEN1 syndrome. In MEN1 patients, insulinomas are often multiple and other manifestations of this syndrome must be considered, although in our patient, due to normal level of serum prolactin, MEN1 was excluded. Insulinoma is a very rare tumor in childhood and adolescence which presents usually with neurologic and sometimes psychiatric signs and symptoms. Most insulinomas have been reported in adults. Whipple's triad is a fundamental diagnostic criteria, although it has some limitation in pediatric patients. Symptoms of hypoglycemia such as headache, palpitation, and confusion mostly happen before meal and during fasting or exercise. Convulsions and episodes of unconsciousness are frequent symptoms that may be followed by drowsiness, behavioural changes, mental deterioration, and ataxia [2–5]. As our patient, neuropsychiatric manifestations are sometimes confusing and can cause delay in diagnosis. Most insulinomas are benign and less than 10% show malignant behaviour and majority of them occur in adults [3, 6]. Early diagnosis of insulinoma is obviously important for ensuring proper treatment and preventing serious adverse neurological consequences and permanent damage [7]. Insulin assay can be helpful in diagnosis; hypoglycemia (BS < 50 mg/dL) with high level of insulin (serum insulin level > 2 mIU/L) or insulin-to-glucose ratio higher than 0.4 in conjunction with high level of C-peptide can lead us to further study [2, 8]. The detection of raised plasma insulin levels at the time of hypoglycemia is the most useful single diagnostic guide [2]. In differential diagnosis of insulinoma, persistent hyperinsulinemic hypoglycemia of infancy (PHHI) and factitious hyperinsulinism must be considered, although PHHI occurs in neonatal period and infancy and so did not match with our patient. On the other hand, in factitious hyperinsulinism, high level of serum insulin is associated with low level of C-peptide. In the mentioned case, C-peptide was not suppressed, so drug-induced hyperinsulinism was excluded.

Most insulinomas are intrapancreatic, benign, and solitary, so localizing study must be used which consists of the following: computed tomography (CT), magnetic resonance imaging (MRI) with gadolinium, octreotide scan, and intraoperative ultrasonography [9]. The most accepted modality of treatment for patient with insulinoma is surgical resection. In the absence of preoperative localization and failed intraoperative detection of an insulinoma, blind pancreatic resection is not recommended [4]. Preoperative tumor localization may require many imaging modalities for avoiding unsuccessful blind pancreatectomy. Surgical methods for resection of insulinoma consist of the following: enucleation for small size lesions and various type of pancreatectomy where, in malignant cases, total pancreatectomy and lymph node dissection may be indicated. At the present time, laparoscopic pancreatectomy has been used with good final results. In our patient, spiral abdominal CT scan revealed a mass at the neck of pancreas but, according to large dimension of tumor, the surgeon had to do a subtotal pancreatectomy, and more limited procedures were not possible. As mentioned before, surgical resection is the only radical method for treatment of insulinoma, but diazoxide and octreotide have been used as medical treatment for suppression of insulin secretion [3]. Pathologic diagnosis of insulinoma may need some complementary IHC studies such as chromogranin A or Ki-67; in our patient, chromogranin A was positive (Figure 5).

## 4. Conclusion

Hyperinsulinism is a rare cause of hypoglycemia in childhood and adolescence and must be evaluated properly.
